# Capturing phenotypic heterogeneity in MPS I: results of an international consensus procedure

**DOI:** 10.1186/1750-1172-7-22

**Published:** 2012-04-23

**Authors:** Minke H de Ru, Quirine GA Teunissen, Johanna H van der Lee, Michael Beck, Olaf A Bodamer, Lorne A Clarke, Carla E Hollak, Shuan-Pei Lin, Maria-Verónica Muñoz Rojas, Gregory M Pastores, Julian A Raiman, Maurizio Scarpa, Eileen P Treacy, Anna Tylki-Szymanska, J Edmond Wraith, Jiri Zeman, Frits A Wijburg

**Affiliations:** 1 Department of Paediatrics, Academic Medical Center, University Hospital of Amsterdam, H7-270, Meibergdreef 9, 1105 AZ Amsterdam, The Netherlands; 2 Department of Paediatric Clinical Epidemiology, Academic Medical Center, University Hospital of Amsterdam, Amsterdam, The Netherlands; 3 Children's Hospital, University Medical Center, Mainz, Germany; 4 Department of General Paediatrics and Neonatology, University Children's Hospital, Vienna, Austria. Current affiliation: Dr. John T. Macdonald Foundation, Department of Human Genetics, University of Miami Miller School of Medicine, Miami, FL, USA; 5 Department of Medical Genetics, University of British Columbia, Vancouver, BC, Canada; 6 Department of Internal Medicine, Division of Endocrinology and Metabolism, Academic Medical Center, University Hospital of Amsterdam, Amsterdam, The Netherlands; 7 Division of Genetics, Department of Paediatrics, Mackay Memorial Hospital, Taipei, Taiwan; 8 Medical Genetics Service, Hospital de Clinicas, Porto Alegre, Brazil. Current affiliation: Genzyme do Brazil, São Paulo, SP, Brazil; 9 Neurogenetics Division, Departments of Neurology and Pediatrics, New York University School of Medicine, New York, USA; 10 Division of Clinical and Metabolic Genetics, Hospital for Sick Children and University of Toronto, Toronto, ON, Canada; 11 Department of Paediatrics, University of Padova, Padova, Italy; 12 National Centre for Inherited Metabolic Diseases, Children's University Hospital, Dublin, Ireland; 13 Department of Metabolic Diseases, The Children's Memorial Health Institute, Warsaw, Poland; 14 Willink Biochemical Genetics Unit, Royal Manchester Children's Hospital, Manchester, UK; 15 Department of Paediatrics, First Faculty of Medicine, Charles University in Prague and General University Hospital, Prague, Czech Republic

**Keywords:** Mucopolysaccharidosis type I, Iduronidase, Classification, Consensus, Phenotype, Hematopoietic stem cell transplantation

## Abstract

**Background:**

Mucopolysaccharidosis type I (MPS I) is traditionally divided into three phenotypes: the severe Hurler (MPS I-H) phenotype, the intermediate Hurler-Scheie (MPS I-H/S) phenotype and the attenuated Scheie (MPS I-S) phenotype. However, there are no clear criteria for delineating the different phenotypes. Because decisions about optimal treatment (enzyme replacement therapy or hematopoietic stem cell transplantation) need to be made quickly and depend on the presumed phenotype, an assessment of phenotypic severity should be performed soon after diagnosis. Therefore, a numerical severity scale for classifying different MPS I phenotypes at diagnosis based on clinical signs and symptoms was developed.

**Methods:**

A consensus procedure based on a combined modified Delphi method and a nominal group technique was undertaken. It consisted of two written rounds and a face-to-face meeting. Sixteen MPS I experts participated in the process. The main goal was to identify the most important indicators of phenotypic severity and include these in a numerical severity scale. The correlation between the median subjective expert MPS I rating and the scores derived from this severity scale was used as an indicator of validity.

**Results:**

Full consensus was reached on six key clinical items for assessing severity: age of onset of signs and symptoms, developmental delay, joint stiffness/arthropathy/contractures, kyphosis, cardiomyopathy and large head/frontal bossing. Due to the remarkably large variability in the expert MPS I assessments, however, a reliable numerical scale could not be constructed. Because of this variability, such a scale would always result in patients whose calculated severity score differed unacceptably from the median expert severity score, which was considered to be the 'gold standard'.

**Conclusions:**

Although consensus was reached on the six key items for assessing phenotypic severity in MPS I, expert opinion on phenotypic severity at diagnosis proved to be highly variable. This subjectivity emphasizes the need for validated biomarkers and improved genotype-phenotype correlations that can be incorporated into phenotypic severity assessments at diagnosis.

## Background

Mucopolysaccharidosis type I (MPS I; OMIM #252800) is a rare autosomal recessive lysosomal storage disorder caused by a deficiency in the alpha-L-iduronidase (IDUA) enzyme, which is involved in the breakdown of the glycosaminoglycans (GAGs) heparan sulfate and dermatan sulfate [1]. The resulting GAG accumulation leads to cellular and organ dysfunction. More than 140 different mutations in the *IDUA *gene have been described [2,3]. The birth prevalence varies from 1 in 26,000 (in the Irish Republic) to less than 1 in 900,000 (in Taiwan) [4,5].

MPS I is traditionally divided into three phenotypes: the severe Hurler (MPS I-H) phenotype, the intermediate Hurler-Scheie (MPS I-H/S) phenotype, and the attenuated Scheie (MPS I/S) phenotype. In reality, however, MPS I presents with a continuous spectrum of phenotypic severity [1,6,7].

MPS I-H patients have marked cognitive delay, coarse facial features, corneal clouding, hearing impairment, ear-nose-throat infections, hepatosplenomegaly, umbilical and inguinal hernias, restricted joint mobility and orthopedic, cardiac and respiratory problems in early childhood. Without appropriate treatment, their life expectancy is severely limited. At the other end of the spectrum, MPS I-S patients have normal intelligence and near-normal life expectancy but experience significant morbidity as a consequence of restricted joint mobility, carpal tunnel syndrome, skeletal dysplasia, cardiac disorders and pulmonary dysfunction. Patients with the intermediate MPS I-H/S phenotype are generally described as having no or only mild cognitive impairment and relatively severe somatic symptoms that limit life expectancy to the 2^nd ^or 3^rd ^decade in the absence of treatment [1,6-9].

Two therapeutic options are currently available: hematopoietic stem cell transplantation (HSCT) and intravenous enzyme replacement therapy (ERT). HSCT can stabilize neurocognitive function, prevent fatal cardio-pulmonary complications, and improve overall survival [10]. However, HSCT can only preserve cognitive function if successfully performed at an early age [10-12], before the onset of significant central nervous system involvement, and it still carries a considerable risk of procedure-related morbidity and mortality. Weekly ERT with recombinant IDUA (laronidase) can improve the respiratory and cardiac symptoms and some of the skeletal and joint manifestations, reduce hepatosplenomegaly, and improve the overall quality of life [13-17]. Because intravenously administered laronidase does not cross the blood-brain barrier [18], ERT cannot prevent cognitive decline in patients with MPS I-H. Therefore, HSCT is the preferred treatment strategy for patients with a presumed MPS 1-H phenotype who are diagnosed before the age of approximately 2.5 years, while patients with the MPS I-H/S and MPS I-S phenotypes may benefit significantly from ERT [12].

When an MPS I diagnosis is made, the phenotype needs to be assessed as soon as possible so that the optimal treatment strategy can be quickly determined. The outcome of HSCT in MPS I-H is less favorable when there is a longer delay between diagnosis and transplant [19,20]. If ERT is the treatment of choice, it probably should be initiated early to prevent irreversible damage [12,21]. Because the predictive value of genotyping is still limited, the phenotypic severity often needs to be assessed solely on the basis of clinical signs and symptoms. However, clear criteria for delineating the different phenotypes are lacking, particularly at the time of diagnosis.

The aim of this study was to develop a consensus scale for phenotypically classifying MPS I patients at diagnosis based on clinical signs and symptoms.

## Methods

The process for constructing a diagnostic disease severity scale included two written rounds, a consensus meeting and a combined modified Delphi method and nominal group technique [22,23]. All of the 16 internationally recognized MPS I experts who were invited to join this project agreed to collaborate. In the first written round, the experts were asked to assess the relative importance of potential criteria for classifying disease severity. For this purpose, a list of 30 criteria selected from a review of the literature was composed by three experts (CEH, FAW and JEW) (Table 1 top panel). The participants rated the 30 criteria (major, intermediate, minor or redundant) according to their perceived importance for phenotypic classification at diagnosis and also proposed additional criteria. Nine additional criteria were suggested (Table 1, bottom panel). Based on these results, seven items that were rated of major importance by the majority of the experts and not rated as redundant by any were selected ('results, first written round', Table 2).

**Table 1:** The item-pool used for developing the MPS I phenotype severity scale

Items	Rated to be of major importance by ≥50% of experts and as 'redundant' by none	Not present in any of the 20 patient descriptions
*Included in first written round:*		
1. Age at onset of symptoms (historical)	x	
2. Age at diagnosis		
3. Coarse facial features		
4. Abdominal hernia		
5. Inguinal hernia		
6. Developmental delay	x	
7. Cognitive decline	x	x
8. Hydrocephalus	x	
9. Cervical cord compression		x
10. Carpal tunnel syndrome		
11. Corneal clouding		
12. Elevated ocular pressure		x
13. Recurrent upper airway infections		
14. Recurrent otitis media		
15. Hearing disorders		
16. Pulmonary function		
17. Respiratory insufficiency		x
18. Obstructive sleep apnea syndrome	x	
19. Valvular heart disease		
20. Cardiomyopathy		
21. Coronary artery disease		x
22. Hepatosplenomegaly		
23. Dysostosis multiplex		
24. Kyphosis	x	
25. Hip dysplasia		
26. Toe walking		x
27. Joint stiffness/arthropathy/contractures	x	
28. Growth retardation		
29. Premature death		x
30. Dermal melanocytosis		x
*Proposed as additional items by the respondents:*		
31. Positive family history		x
32. Hypertrichosis		
33. Macroglossia		
34. Hypotonia in infancy		
35. Protruded sternum		
36. Large head/frontal bossing		
37. Psychosis		x
38. Affected sibling		x
39. Early accelerated growth		x

**Table 2:** An overview of results during the different stages of the consensus procedure

	'results, 1^st ^written round' Considered to be of major importance by the majority and redundant by none of the experts	'results, 2^nd ^written round' Resulting from statistical comparisons of the median expert scores	'results, consensus meeting' Final consensus list
Cognitive decline	x		
Age at onset of symptoms	x	x	x
Developmental delay	x	x	x
Hydrocephalus	x		
Kyphosis	x	x	x
Obstructive sleep apnea syndrome	x		E
Joint stiffness/arthropathy/contractures	x		x
Large head/frontal bossing		x	x
Early diagnosis		x	
Dysostosis multiplex		x	E
Coarse facial features		x	
Cardiomyopathy			x
Growth retardation			E

E: excluded from the final severity scale after working group discussions

In the second written round, 20 MPS I case descriptions were sent to the experts. The case descriptions were based on clinical information available at the time of diagnosis and were retrieved from two participating centers (Amsterdam and Manchester). The cases were selected to represent a wide range of MPS I phenotypes. The experts rated each case description for severity on an 11-point scale (0 = mildest; 10 = most severe). If one of the experts was involved in treating one of the specific cases, that expert's rating for that case was excluded from the analyses. The median expert rating for a case was considered to be the 'gold standard' severity score.

All 20 case descriptions were scored by one investigator (QGAT) according to the presence or absence of the 39 criteria in Table 1. Mann-Whitney U tests were used to investigate the associations between the median expert case scores and the presence or absence of each of the 39 items. An item was considered to be "key" when there was a significant difference (p < 0.05) between the median expert rating of the cases for which the item was present and the median expert rating of the cases for which the item was not present. The list of key items resulting from this analysis constituted the 'results, second written round' (Table 2).

A face-to-face consensus meeting was subsequently held in Amsterdam (in May 2008). To investigate the intra-observer reproducibility [24], all 20 case descriptions were presented in a random order and rated by the experts for severity again. The experts were then informed of the results of the two written rounds, and all of the case descriptions were then separately discussed. The experts were asked to explain which items they considered to be most important for assessing disease severity in a particular case, which led to discussions about which of the items were most important and how they should be defined. Next, every expert proposed a list of the items they deemed important for a severity scale. These items were ranked according to the frequency with which they had been proposed (Table 3) and compared with the items in the 'results, second written round' (Table 2). Finally, a consensus was reached on the list of items considered to be most important for phenotypic classification ('results, consensus meeting'; Table 2).

**Table 3:** The 24 items that were proposed by the individual experts during the meeting

Items	Number of experts who proposed each item^#^
1. Global developmental delay/cognitive decline	18
2. Age at onset of symptoms <1.5 yr	13
3. Joint stiffness/arthropathy/contractures	11
4. Kyphosis/spinal involvement	11
5. Dysostosis multiplex	10
6. Cardiac involvement: valvular disease + cardiomyopathy	10
7. Large head/frontal bossing	8
8. Hydrocephalus	7
9. Age at diagnosis <1.5 yr	7
10. Coarse facial features	6
11. Growth retardation	4
12. Hepatosplenomegaly	4
13. Corneal clouding	4
14. Pulmonary function	4
15. Obstructive sleep apnea syndrome	3
16. Hearing problems	3
17. Cervical cord compression	2
18. Carpal tunnel syndrome	2
19. Abdominal hernia	1
20. Hip dysplasia	1
21. Recurrent otitis media	1
22. Macroglossia	1
23. Early surgical intervention	1
24. Affected sibling	1

# Certain items were individually mentioned by the experts but were used as a single item in the sum score (for example, developmental delay and cognitive decline were counted together); such items explain why the number of experts proposing a certain item occasionally exceeds the total number of experts

For the three items in which the severity definition or grading was complicated, working groups of 3-5 experts were asked to make a proposal for each item based on the best evidence.

After the meeting, the scores of the items in the final consensus list that were present in each patient description were equally weighted and summed to obtain an objective MPS I phenotypic severity score at diagnosis. The correlation between the scores on this 'MPS I severity scale' and the median expert scores ('gold standard') for the 20 case descriptions was calculated.

To assess the validity of this scale, a set of 18 new MPS I case descriptions, again representing the full MPS I phenotypic spectrum, was compiled. Care was taken to include information on all six of the items in the final consensus list (Table 2) in each case description. The experts rated the new cases for severity (0-10), and the correlation between the objective phenotypic severity scores and the median expert scores for the 18 case descriptions was calculated.

### Statistical analysis

The association between the presence or absence of each item and the median expert severity scores for the individual cases was investigated using the Mann-Whitney *U *test (SPSS 18.0). To quantify the intra- and inter-rater reproducibility of the expert scores, the variance components were calculated using a three-way random effects analysis of variance model with the scores as the dependent variable. The following variance components were estimated using the model: between patients (σ_P_^2^), between experts (σ_E_^2^), between rounds (σ_R_^2^), the patients × experts interaction (σ_PE_^2^), the patients x rounds interaction (σ_PR_^2^), the experts × rounds interaction (σ_ER_^2^), and the residual (σ_PER_^2^). Because the absolute values of the expert scores were not of interest, intraclass correlation coefficients (ICCs) were calculated to assess consistency [25].

The intra-rater ICC_consistency _was calculated using the following formula:

$$\text{ICC}=\frac{{\sigma^{\text{2}}}_{\text{P}}+{\sigma^{\text{2}}}_{\text{PE}}}{{\sigma^{\text{2}}}_{\text{P}}+{\sigma^{\text{2}}}_{\text{PE}}+{\sigma^{\text{2}}}_{\text{PR}}+{\sigma^{\text{2}}}_{\text{PER}}}$$

The inter-rater ICC_consistency _was calculated as follows:

$$\text{ICC}=\frac{{\sigma^{\text{2}}}_{\text{P}}+{\sigma^{\text{2}}}_{\text{PE}}}{{\sigma^{\text{2}}}_{\text{P}}+{\sigma^{\text{2}}}_{\text{PE}}+{\sigma^{\text{2}}}_{\text{PR}}+{\sigma^{\text{2}}}_{\text{PER}}}$$

There was no 'rounds' factor in the inter-rater reproducibility of the subjective expert scores of the 18 patient descriptions, which therefore use the following formula:

$$\text{ICC}=\frac{{\sigma^{\text{2}}}_{\text{P}}}{{\sigma^{\text{2}}}_{\text{P}}+{\sigma^{\text{2}}}_{\text{PE}}}$$

Spearman correlation coefficients were used to investigate correlations between the median expert scores and the objective phenotypic severity scores. Numerical weights were assigned to the items used in the objective score according to the experts' assessment of their clinical relevance. The goal was to maximize the correlation with the median subjective expert score and thus achieving the best phenotypic differentiation. The level of statistical significance was set at p < 0.05.

## Results

**Figure 1:**
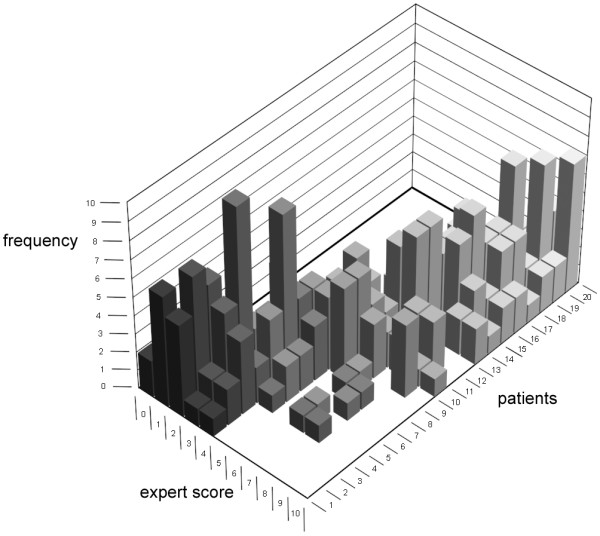
**The expert scores per patient (for the 20 case descriptions in the second written round, in order of ascending median expert score)**.

Of the 30 items included in the first-round questionnaire (Table 1), 7 were scored as being of ' major importance' by ≥ 50% of the experts and as 'redundant' by none of the experts (Table 2). Nine additional items were proposed (Table 1). The median expert scores of the 20 case descriptions used in the second written round ranged from 1 to 9. There was considerable variation in the expert scores for each case description (Figure 1). The difference between the highest and lowest expert case scores ranged from 2 (for a case with a median expert score of 3) to 8 points (for a case with a median expert score of 6). The intra- and inter-observer ICCs for the expert scores were 0.79 and 0.75, respectively.

Of the 39 items identified in the 'first written round' (Table 1), 12 were not found in any of the 20 case descriptions. The case scores obtained by summing the remaining 27 items (with an item scoring 1 if it was present and 0 otherwise) ranged from 4 to 18. The frequencies of the 27 items in the 20 case descriptions ranged from 5% to 90% (Table 4). The correlation between the case scores from the 27 items and the median expert scores was 0.61, p = 0.004. A significant difference in median expert scores was found between the groups determined by the presence or absence of 7 items ('results, second written round', Table 2): 1) age at onset of symptoms (<1.5 yr) (median difference 4, p = 0.015); 2) developmental delay (median difference 4.5, p = 0.002); 3) kyphosis (median difference 5, p = 0.001); 4) large head/frontal bossing (median difference 4, p = 0.037); 5) early diagnosis (<1.5 yr) (median difference 4, p = 0.001); 6) dysostosis multiplex (median difference 4, p < 0.001) and 7) coarse facial features (median difference 3.5, p = 0.012). The correlation between the unweighted sum score of these seven items and the median expert score was 0.91; p < 0.001.

**Table 4:** The 27 items occurring in the 20 case histories, in ascending order of frequency

Items	Frequency of occurrence (%)
1. Disturbed pulmonary function	5
2. Hypertrichosis	5
3. Sternum protruded	5
4. Cardiomyopathy	10
5. Macroglossia	10
6. Growth retardation	15
7. Hypotonia in infancy	15
8. Hydrocephalus	20
9. Carpal tunnel syndrome	25
10. OSAS	25
11. Hip dysplasia	30
12. Large head/frontal bossing*	35
13. Valvular heart disease	40
14. Early diagnosis (<1.5 yr)*	40
15. Inguinal hernia	40
16. Hearing problems	40
17. Developmental delay*	55
18. Dysostosis multiplex*	55
19. Kyphosis*	65
20. Abdominal hernia	70
21. Coarse facial features*	70
22. Early onset of symptoms (<1.5 yr)*	75
23. Joint stiffness/arthropathy/contractures	80
24. Hepatosplenomegaly	75
25. Corneal clouding	85
26. Recurrent otitis media	85
27. Recurrent upper airway infection	90

For the items marked with an*, there was a statistically significant difference in the median expert score between the cases with and without this item.

The 24 items proposed by the individual experts are shown in Table 3 in order of descending frequency. During the meeting, these 24 items were compared with the 7 items from the 'results, second written round' (Table 2). Consensus was reached on including nine of the items in the list of those considered most important for phenotypically classifying MPS I patients (Table 2, 'results, consensus meeting'). For three items, additional discussion was considered necessary in separate working groups after the meeting. Within several weeks after the consensus meeting, the working groups proposed deleting three items from the final consensus list: 1) dysostosis multiplex, 2) growth retardation, and 3) obstructive sleep apnea syndrome (OSAS). The main reason for the exclusions was the inability of the items to differentiate between the phenotypes. All experts agreed with deleting these three items from the final consensus list.

**Figure 2:**
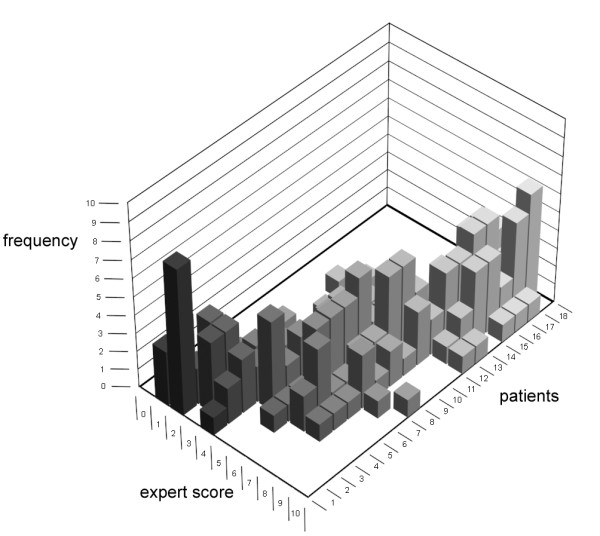
**The expert scores per patient (for the 18 case descriptions in the validation round, in order of ascending median expert score)**.

The median expert scores of the 18 case descriptions used in the validation phase ranged from 2 to 8.5. The inter-observer ICC of the expert scores was 0.71. Again, there was considerable variation in the expert severity assessments of the case descriptions (Figure 2). The differences between the highest and lowest expert scores ranged from 3 (for 7 cases with median expert scores of 2.0, 2.5, 7.0, 8.0, 8.0, 8.5 and 8.5) to 8 points (for a case with a median expert score of 7.0).

Despite the reasonable correlations between both the weighted and unweighted sum scores of the six items in the 'final consensus list' and the median expert scores, it became clear that there would always be patients whose calculated severity score differed unacceptably from the median expert severity score, which was considered the 'gold standard'. For this reason, and because of the considerable variability between the experts, we decided to refrain from presenting the six criteria in a numerical 'severity scale'.

## Discussion

This consensus procedure was designed to develop a numerical scale for assessing MPS I phenotypic severity at diagnosis to facilitate treatment decisions and patient communication.

Consensus was reached on a list of six items that were considered to be most important for phenotypically classifying MPS I patients at diagnosis (Table 2, 'final consensus list'). Our consensus procedure also identified several items generally considered to be major hallmarks of MPS I that were nevertheless excluded from the list of key items for the following reasons. First, the signs and symptoms important for establishing an MPS I diagnosis often do not differentiate between mild and severe phenotypes, e.g., umbilical or inguinal hernias, coarse facial features, and the presence or severity of corneal clouding and hepatosplenomegaly. Second, although some symptoms (e.g., hydrocephalus) are frequently encountered in young patients with severe phenotypes, the experts did not consider their absence to be an indicator of a mild phenotype. The 'diagnosis at a young age' item was also excluded because it can be influenced by several factors, such as diagnostic difficulties or delays in seeking medical attention. The age of onset of any of the six key signs and symptoms was considered to be a more valuable indicator of phenotypic severity. Finally, cognitive decline was not included as a key item because this item can only be assessed during follow-up (in contrast to developmental delay, which can usually be ascertained at the time of diagnosis).

As a result of the process of item generation, selection and validation, we decided that constructing a reliable numerical scale for assessing phenotypic severity in MPS I patients was not feasible due to the remarkable variability in the expert assessments (Figures 1 and 2), which resulted in a large inter-observer variability. As a result, the expert score that served as the 'gold standard' proved to be unreliable. Even in the patients with high median expert scores, indicating a severe phenotype, the individual expert scores varied considerably, with some experts also assigning the cases intermediate scores (Figures 1 and 2). As a result of this variability among the expert assessments, there would always be patients (even those with the most severe MPS I-H phenotype) whose severity scores from a system based on the six selected items would differ considerably from the median expert score and from a severity score given by several of the experts.

Comparably significant variability in expert severity assessment will likely also occur for other rare diseases, including other lysosomal storage diseases, in which there are pleiotropic and progressive disease manifestations. Although the methods applied in our study may be used to tease out factors related to assessing disease severity that are comparable to the six key items that we obtained for MPS I (Table 2), constructing a reliable severity scale based on clinical signs and symptoms may often be impossible.

The need for reliable and early prediction of MPS I phenotypic severity has become even more pressing with the development of high-throughput newborn screening (NBS) techniques based on measuring IDUA activity and/or immune-quantification of the IDUA protein in dried blood spots [26-28]. A severity score based on clinical signs and symptoms will certainly not be useful in this context, given that a number of signs and symptoms will not be present in the neonatal period.

Because clinical signs and symptoms appear to be insufficiently reliable to assess phenotypic severity at diagnosis, other methods should be vigorously investigated. Combined genotyping and biomarker analysis in plasma and/or urine, such as the recently reported plasma heparin cofactor II-thrombin (HCII-T) complex and the urinary dermatan sulfate:chondroitin sulfate (DS:CS) ratio, promises to be a good strategy for determining disease severity in newly diagnosed MPS I patients [2,3,29,30]

Our study has several limitations. First, the experts differed with respect to the age, phenotype and ethnic background of their patient experiences. These observations may have influenced their opinion of disease severity. Second, the patient information used to write the case descriptions was gathered retrospectively. Thus, some follow-up results were known for most of the patients, which may have biased the data retrieval and the description of the cases. Moreover, the information that had been recorded in the patient files may have been influenced by knowledge of which interventions had (or had not) been performed. Finally, assessing phenotypic severity is hampered by the subjective rating of certain items, e.g., the presence or absence of kyphosis and frontal bossing on clinical examination, the parents' report of the age of symptom onset and the influence of decreased range of motion due to joint disease on performing activities of daily living.

## Conclusions

This robust and transparent consensus procedure did not result in a reliable and validated numerical MPS I severity scale. However, the process did produce a list of six items rated by the experts as being most important for phenotypic classification, which may be useful for classifying newly diagnosed MPS I patients. Further studies into the possible roles of genotypes and biomarkers as indicators of disease severity are necessary to optimize clinical diagnosis and decision-making in MPS I patients.

## Abbreviations

ERT: Enzyme replacement therapy; GAG: Glycosaminoglycan; HSCT: Hematopoietic stem cell transplantation; ICC: Intraclass correlation coefficient; IDUA: Alpha-L-iduronidase; MPS I: Mucopolysaccharidosis type I; NBS: Newborn screening; OSAS: Obstructive sleep apnea syndrome.

## Competing interests

The authors declare that they have no competing interests.

## Authors' contributions

FAW, CEH, JHL, JEW and QGAT were part of the planning committee, were involved in the methodological process, and wrote the first draft of the manuscript. MHdR was involved in the validation phase and wrote later drafts of the manuscript. JHL performed the statistical analysis. ATS, CEH, EPT, FAW, GMP, JAR, JEW, JZ, LAC, MB, MS, MVMR, OAB, and SPL participated in written rounds 1 and 2 and the face-to-face consensus meeting. All of the authors reviewed and corrected earlier versions of the manuscript and participated in the creation of its final form.
